# Prospective evaluation of the lymph node proteome in dogs with multicentric lymphoma supplemented with sulforaphane

**DOI:** 10.1111/jvim.15898

**Published:** 2020-09-14

**Authors:** Cyril Parachini‐Winter, Shay Bracha, Stephen A. Ramsey, Liping Yang, Emily Ho, Haley J. Leeper, Kaitlin M. Curran

**Affiliations:** ^1^ Department of Clinical Sciences Carlson College of Veterinary Medicine, Oregon State University Corvallis Oregon USA; ^2^ Department of Biomedical Sciences School of Electrical Engineering and Computer Science, Oregon State University Corvallis Oregon USA; ^3^ Department of Chemistry College of Science, Oregon State University Corvallis Oregon USA; ^4^ Linus Pauling Institute and College of Public Health and Human Sciences Oregon State University Corvallis Oregon USA

**Keywords:** cruciferous vegetables, epigenetic, immunity, isothiocyanate, lymphoma and lymphosarcoma, proteome

## Abstract

**Background:**

Lymphoma (LSA) is a common malignancy in dogs. Epigenetic changes are linked to LSA pathogenesis and poor prognosis in humans, and LSA pathogenesis in dogs. Sulforaphane (SFN), an epigenetic‐targeting compound, has recently gained interest in relation to cancer prevention and therapy.

**Objective:**

Examine the impact of oral supplementation with SFN on the lymph node proteome of dogs with multicentric LSA.

**Animals:**

Seven client‐owned dogs with multicentric LSA.

**Methods:**

Prospective, nonrandomized, noncontrolled study in treatment‐naïve dogs with intermediate or large cell multicentric LSA. Lymph node cell aspirates were obtained before and after 7 days of oral supplementation with SFN, and analyzed via label‐free mass spectrometry, immunoblots, and Gene Set Enrichment Analysis.

**Results:**

There was no clinical response and no adverse events attributed to SFN. For individual dogs, the expression of up to 650 proteins changed by at least 2‐fold (range, 2‐100) after supplementation with SFN. When all dogs where analyzed together, 14 proteins were significantly downregulated, and 10 proteins were significantly upregulated after supplementation with SFN (*P* < .05). Proteins and gene sets impacted by SFN were commonly involved in immunity, response to oxidative stress, gene transcription, apoptosis, protein transport, maturation and ubiquitination.

**Conclusions and Clinical Importance:**

Sulforaphane is associated with major changes in the proteome of neoplastic lymphocytes in dogs.

**Abbreviations**
14‐3‐3‐θ: 14‐3‐3 protein theta
AEs: adverse events
D0: day 0
D7: day 7
DNMT: DNA methyltransferase
DNMTi: DNA methyltransferase inhibitor
FC: fold change
FDR: false discovery rate
FNA: fine needle aspiration
GSEA: gene set enrichment analysis
HDAC: histone deacetylase
HDACi: histone deacetylase inhibitor
HIP: Hsc70‐interacting protein
HRP: horseradish peroxidase
LC‐MS/MS: liquid chromatography and tandem mass spectrometry
LSA: lymphoma
NER: normalized expression ratio
NRF2: nuclear factor E2‐related factor 2
RI: reference interval
SFN: sulforaphane
VCOG: Veterinary Cooperative Oncology Group

## 1 INTRODUCTION

The prognosis for dogs with multicentric lymphoma (LSA) has not substantially improved in the last 2 decades. The mainstay of treatment is multiagent chemotherapy, but most dogs eventually die from the disease within 6 to 12 months after diagnosis.^1^, ^2^, ^3^, ^4^ With the addition of radiation therapy or immunotherapy, no major improvement in the prognosis is noted when compared to chemotherapy alone.^5^, ^6^, ^7^, ^8^ Therefore, new treatments avenues for dogs with multicentric LSA are warranted.

Sulforaphane (SFN) is an isothiocyanate derived from cruciferous vegetables such as broccoli or cauliflower.^9^, ^10^ Sulforaphane has chemopreventive activity in multiple cancer types.^9^ One of the main mechanism of action of SFN is to promote the dissociation of nuclear factor E2‐related factor 2 (Nrf2) from the cytoplasmic protein Keap1, with subsequent nuclear translocation of Nrf2 and modulation of many antioxidant response element‐driven genes.^9^, ^10^, ^11^ In this manner, SFN inhibits phase 1 enzymes (ie, cytochrome P450), which convert procarcinogens into carcinogens. It also induces several phase 2 enzymes (ie, glutathione transferase), which detoxify carcinogens and facilitates their excretion from the body.^9^

Epigenetic alterations refer to changes in gene expression and heritable traits that do not involve modifications of the DNA sequence.^12^ For example, removal of acetyl groups from the histone tails by histone deacetylases (HDAC) results in a closed chromatin conformation and repression of gene transcription.^13^ The addition of methyl groups in the promoter regions of genes by DNA methyltransferases (DNMT) interferes with the binding of transcription factors and leads to gene silencing.^12^ Sulforaphane, in addition to its chemopreventive properties, also caries cancer‐suppressive properties by acting as a HDAC inhibitor (HDACi) and DNMT inhibitor (DNMTi) in various cancer cells, leading to re‐expression of various tumor suppressor genes.^9^, ^14^, ^15^, ^16^, ^17^, ^18^, ^19^, ^20^, ^21^ Secondary to its effects on the epigenome or by direct SFN‐protein interactions, SFN also induces major changes in the proteomic profile of several cancer cells in rodents and people.^22^, ^23^, ^24^

Since epigenetic events are reversible, various agents targeting the epigenome have been developed for people with LSA.^25^, ^26^, ^27^ On the other hand, although there is evidence for the presence of epigenetic dysregulations in dogs with LSA,^28^, ^29^, ^30^, ^31^, ^32^, ^33^ little research has been performed regarding the use of epigenetic‐targeted treatments. A bioavailability profile of SFN in healthy dogs is comparable to humans, with a significant decrease in HDAC activity observed 24 hours after consumption of 1 dose of SFN.^34^ Sulforaphane also reduces cell invasion and decreases focal adhesion kinase phosphorylation in canine osteosarcoma cell lines.^35^ However, to our knowledge, the impact of SFN in cancer‐bearing dogs has not been explored.

The primary objective of this study was to analyze changes in the lymph node proteome of dogs with treatment‐naïve multicentric LSA before and after supplementation with SFN. A secondary objective was to assess clinical response and adverse events (AEs) associated with supplementation with SFN alone.

## 2 MATERIALS AND METHODS

### 2.1 Enrollment

This prospective, nonrandomized, noncontrolled study was carried out with approval of the Oregon State University Institutional Animal Care and Use Committee. Dogs were screened for inclusion at the Oregon State University Carlson College of Veterinary Medicine. To be enrolled, each dog had to be newly diagnosed with multicentric LSA by cytology or histopathology, flow cytometry performed for immunophenotyping, a body weight > 15 kg, be clinically healthy at diagnosis (substage a), adequate organ function (absolute neutrophil count >1500 cells/μL; hematocrit >25%; platelets >75 000/μL; creatinine <2× the upper limit of reference interval [RI]; bilirubin ≤1.5× the upper limit of RI; ALT ≤3× the upper limit of RI), a Veterinary Cooperative Oncology Group (VCOG) performance status <2,^36^ and at least 2 lymph nodes >2 cm longest diameter. Exclusion criteria included dogs that had received any previous treatment for lymphoma including corticosteroids, concurrent malignancy or other serious systemic disorder, and dogs administered homeopathic/alternative therapies within 3 days of enrollment (in particular any food, vegetable treats or supplements containing cruciferous vegetables). All screening tests occurred within 1 week of enrollment, and signed informed consent was obtained from all owners before study enrollment.

### 2.2 Study design and sample processing

On day 0 (D0), lymph node fine needle aspirates (FNA) were collected. A total of 3 FNAs were obtained from at least 2 different peripheral lymph nodes from each dog. When possible, collection from mandibular lymph nodes was avoided. All dogs subsequently received 3 capsules of BroccoMax (Jarrow Formulas, Los Angeles, CA) twice daily by mouth for 7 days. BroccoMax is a commercially available broccoli seed supplement containing 30 mg of glucoraphanin per capsule, which yield approximately 8 mg of SFN after conversion by the enzyme myrosinase.^34^ This dose was based on the bioavailability study performed by our research team in healthy dogs,^34^ and on a similar dosing regimen utilized in a human breast cancer trial.^37^ The period of supplementation was chosen based on several studies in mice and human suggesting that 1 to 7 days of supplementation was sufficient for the SFN to reach meaningful levels in various tissues, induce major proteomic changes in tumor cells, and impact the tumor burden as a whole in xenograft models.^11^, ^16^, ^20^, ^38^ Owners were directed to feed dogs their usual food, and avoid any treats for the duration of the trial. On day 7 (D7), duplicate lymph node samples were obtained after the last dose of supplementation with SFN. After sample collection on D7, dogs were deemed off‐study and free to pursue mainstay chemotherapy treatment.

At D0 and D7, material obtained from the 3 FNA samples were mixed together at room temperature with 1 mL of 0.9% sterile saline in a cryovial. Immediately after, 400 μL of the lymph node samples and saline mixture were added, at room temperature, to another cryovial with 100 μL of RIPA protein lysis buffer (150 mM NaCl, 1% Triton X‐100, 0.5% sodium deoxycholic acid [NaDOC], 0.1% SDS, 20 mM TRIS pH 8.0). The cryovial was then immediately flash frozen in liquid nitrogen. All cryovials were kept in liquid nitrogen for a maximum of 15 minutes before being stored at −80°C until further analysis.

All AEs occurring during supplementation with SFN were recorded by the owner, in a standardized questionnaire that was sent home on D0 with each dog. At D7, the AEs recorded were graded according to the VCOG common terminology criteria for AEs.^36^ The clinical response to SFN at D7 was assessed via caliper measurement of peripheral lymph nodes. Disease response was assessed according to the VCOG response evaluation criteria.^39^

### 2.3 Mass spectrometry

The protein samples prepared from the matched lymph nodes of dogs at D0 and D7 were analyzed by liquid chromatography and tandem mass spectrometry (LC‐MS/MS). The protein concentration of each sample was determined using Pierce BCA protein assay kit (Thermo Fisher Scientific, Rockford, Illinois). For each sample, 50 μg of proteins were digested by sequencing grade modified trypsin (Promega Corporation, Madison, Wisconsin). Peptide analysis was achieved using an Orbitrap Fusion Lumos mass spectrometer with a Nano ESI source (Thermo Scientific, Waltham, Massachusetts) coupled with a Waters nanoAcquity UPLC system (Waters, Milford, Massachusetts). The proteolytic products were desalted and loaded on a nanoAcquity UPLC 2G Trap Column (180 μm × 20 mm, 5 μm) for 5 minutes with solvent 0.1% formic acid in 3% ACN at a flow rate of 5 μL/min. An nanoAcquity UPLC RPeptide BEH C18 column (100 μm × 100 mm, 1.7 μm) was applied to separate peptides following by a 120‐minutes gradient consisting of 0.1% formic acid in H2O (mobile phase A) and 0.1% formic acid in ACN (mobile phase B), where B was increased from 3% to 10% at 3 minutes, 10% → 30% at 105 minutes, 30% → 90% at 108 minutes and held 4 minutes, and then decreased to 3% at 113 minutes and held until 120 minutes. The LC flow rate was set at 500 nL/min. All mass spectral data were acquired in the positive ion mode. The spray voltage was 2400 V and the ion transfer tube temperature was 300°C. MS and MS/MS spectra were acquired by the Orbitrap analyzer (resolution 120 K at m/z 200) and Ion Trap (collision induced dissociation CID) respectively. Automatic gain control target was set to 4.0 × 10^5^ for precursor ions and 10^4^ for product ions. Mass tolerances were set at ±10 ppm for precursor ions and 0.6 Da for fragment ions.

Raw data files were analyzed with Thermo Scientific Proteome Discoverer 2.2 software and searched against the Uniprot Canis database using Sequest HT as search engine. To allow GO annotation analysis for potential canine protein orthologues, the datasets were also searched against the Uniprot *Homo sapiens* protein database.

To calculate the fold change (FC) of a given protein, each peptide group ratio was first calculated as the geometric median of all combinations of ratios from all the replicates in the same group. The protein ratio was subsequently calculated as the geometric median of the peptide group ratios. The proteins FC between the following groups was investigated: (1) all dogs at D0 vs all dogs at D7 (FC D0/D7); (2) dogs with intermediate/large B‐cell LSA at D0 vs dogs with intermediate/large B‐cell LSA D7 (FC_B_ D0/D7); and (3) each dog individually at D0 vs D7. For all dogs taken together and for comparison within dogs with intermediate/large B‐LSA, only proteins with a significant FC (*P* < .05) were retained. For comparisons within individual dogs before and after SFN, no statistical comparison was performed, and only proteins with a FC < 0.5 or > 2 (at least twice as high or twice as low after SFN) were retained. Finally, only proteins detected in over 50% of samples at each time point or proteins detected at only 1 time point but not the other were selected for further analysis.

### 2.4 Immunoblots

The protein concentration of each sample was assessed using Pierce BCA protein assay kit (Thermo Fisher Scientific). Fifty μg of proteins were loaded per well. Proteins were separated by sodium dodecyl sulfate polyacrylamide gel electrophoresis and transferred onto a nitrocellulose membrane. Membranes were washed with TBS (20 mM Tris‐HCl pH 7.4 and 150 mM NaCl) and blocked overnight at 4°C with Odyssey blocking buffer (LI‐COR, Lincoln, NE). Blots were incubated overnight at 4°C with a goat polyclonal anti‐14‐3‐3‐θ antibody (PA5‐18822, Thermo Fisher Scientific, Rockford, Illinois) diluted 1:5000 with TBST (TBS with 0.1% Tween‐20). Replicate blots were probed with a rabbit polyclonal anti‐Hsc70‐interacting protein (HIP) antibody (NBP2‐47427, Novus Biologicals, Centennial, CO) diluted 1:5000 with TBST. Blots were then washed and incubated for 1 hour at room temperature with the corresponding secondary antibodies: a horseradish peroxidase (HRP)‐labeled donkey anti‐goat polyclonal antibody (sc‐2020, Santa Cruz Biotechnology, Dallas, Texas) diluted 1:1000 in TBST, and a biotin‐SP‐conjugated goat anti‐rabbit polyclonal antibody (AB‐2337959, Jackson ImmunoResearch, West Grove, Pennsylvania) diluted 1:20000 in TBST followed by a HRP‐conjugated streptavidin (Jackson ImmunoResearch) diluted 1:1000 in TBST. Blots were then exposed to luminol and peroxide (ECL Prime Western Blotting Detection Reagent, GE Healthcare Life Sciences, Marlborough, Massachusetts), and visualized using the ImageQuant LAS 4000 scanning system (GE Healthcare Life Sciences).

The β‐actin protein was used to normalize the expression level of 14‐3‐3‐θ and HIP. Blots were incubated for 15 minutes at room temperature with RestorTM PLUS Western Blot stripping buffer (Thermo Fisher Scientific), blocked overnight with Odyssey blocking buffer, and incubated at room temperature for 2 hours with a mouse monoclonal anti‐β‐actin primary antibody (Santa Cruz Biotechnology) diluted 1:200 with TBST. Blots were then incubated at room temperature for 1 hour with a HRP‐labeled goat anti‐mouse secondary antibody (Santa Cruz Biotechnology) diluted 1:5000 with TBST, and revealed with luminol. The volume of HIP, 14‐3‐3‐θ band, and β‐actin bands was determined using the ImageQuant TL 8.2 software (GE Healthcare Life Sciences). The volume of HIP and 14‐3‐3‐θ bands was divided by the volume of the corresponding actin band, yielding a normalized expression for HIP and 14‐3‐3‐θ. The normalized expression ratio (NER) D7/D0 was defined as the normalized expression at D7 divided by the normalized expression at D0.

### 2.5 Gene set enrichment analysis

We used the R statistical computing environment (version 3.3.3) to generate ranked lists of genes from the protein abundance ratio data. First, we mapped canine proteins (identified by UniProtKB accession identifiers) to canine Ensembl protein identifiers using Ensembl BioMart (release 94), and for any identifiers that failed to map by BioMart, we used the Bioconductor (version 3.4) package org.Cf.eg.db (version 3.7.0, which is based on Entrez Gene version 2018‐Oct11) for identifier mapping. Second, we mapped canine Ensembl protein identifiers to human Ensembl protein identifiers using the Bioconductor (version 3.4) package hom.Hs.inp.db (version 3.1.2, which uses the Inparanoid ortholog database version 8.0). Third, we mapped human Ensembl protein identifiers to Entrez gene identifiers using Ensembl BioMart, and for any identifiers that failed to map by BioMart, we secondarily used the Bioconductor (version 3.4) package org.Hs.eg.db (version 3.7.0, which is based on Entrez Gene version 2018‐Oct11). We log_2_‐transformed the protein expression ratios for each protein and programmatically generated a Gene Set Enrichment Analysis (GSEA) ranked gene (.rnk) file containing human Entrez Gene identifiers.

We analyzed the ranked gene files using GSEA (version 3.0) in GSEAPreranked mode, using gene sets H (Hallmarks of Cancer), C2 (Curated Gene Sets), C3 (Regulatory Target Gene Sets), and C5 (Gene Ontology Gene Sets) from the MSigDB database (version 6.2, http://software.broadinstitute.org/gsea/msigdb) and with a minimum set size of 10. Only gene sets with a false discovery rate (FDR) *q* < 0.1 and a nominal *P* < .01 were retained for the final analysis. In this analysis, a FDR < 0.1 indicates that 9 out of 10 gene sets that were found to be significantly up‐ or down‐regulated at D7 would be expected to be validated as significantly up‐ or down‐regulated.

## 3 RESULTS

### 3.1 Demographics, clinical response to sulforaphane and outcome

Seven dogs were prospectively enrolled between March 2017 and March 2018. No dog had a history of systemic disorder or other comorbidity, and all dogs were clinically healthy at the time of enrollment. Breeds included Australian shepherd (n = 2), husky, pitbull, labrador, beagle, and English shepherd (1 each). There were 3 spayed females, 3 neutered males and 1 intact male. Median age at the time of enrollment was 6 years (range, 3‐13). Based on cytology results, all dogs were diagnosed with intermediate or large cell LSA. Based on flow cytometry results, 3 dogs were diagnosed with B‐cell LSA, 2 dogs with T‐cell LSA and 1 dog with large B‐cell LSA with possible concurrent T‐zone LSA.

All but 1 dog developed AEs, for a total of 24 episodes of AEs recorded. All AEs were grade 1 or 2, and most commonly lethargy, vomiting and anemia. More details are provided in Table 1. None of the AEs were directly attributed to SFN. Most were deemed likely related to LSA, as 33% of AEs had been identified prior to the study, and the most severe AEs occurred in dogs experiencing progressive disease while receiving SFN. No SFN dose delays or modification were required. According to the VCOG criteria,^39^ no dogs experienced an objective response. Four dogs had progressive disease and 3 dogs had stable disease.

**TABLE 1:** Summary of adverse events experienced by dogs with multicentric lymphoma during 7 days of oral supplementation with sulforaphane

Adverse event	Grade 1	Grade 2	Grade 3	Grade 4	Grade 5	Total
Lethargy	3	1				4
Anemia	3					3
Vomiting	3					3
Diarrhea	1	1				2
Anorexia	1	1				2
Thrombocytopenia	2					2
Increase ALT	2					2
Increase ALP	2					2
Lameness	1					1
Seroma	1					1
Pain	1					1
Polyuria	1					1

After the end of the study, 6 dogs were treated with cyclophosphamide, vincristine, doxorubicin, prednisone (CHOP) chemotherapy protocol. One dog was treated with L‐asparaginase and prednisone after completion of the study. At the time of writing, 6 dogs had died or were euthanized for lymphoma‐related causes at 42, 153, 247, 338, 344, and 387 days postdiagnosis, and dog #3 is still alive.

### 3.2 Mass spectrometry: All dogs at day 7 vs day 0

A total of 915 proteins were identified from all lymph nodes samples. Statistical analysis discovered 24 proteins with significant FC between D7 and D0 (*P* < .05) Among these, 14 were significantly downregulated at D7 (median FC D7/D0 = 0.32; range, 0.13‐0.48), and 10 were significantly upregulated at D7 (median FC D7/D0 = 3.8; range, 3‐5.3). After searching the raw data against Uniprot Canine and *Homo sapiens* database, 21 of these 24 proteins were precisely identified and the results are presented in Table 2.

**TABLE 2:** Characteristics of 21 proteins significantly downregulated (n = 12) or upregulated (n = 9) after a week of oral supplementation with sulforaphane in 7 dogs with multicentric lymphoma

Protein (*GENE*) downregulated at day 7	FC D7/D0	*P*‐value
tRNA‐splicing ligase RtcB homolog (*RTCB*)	0.13	5.2 × 10 ^−11^
Lamin A/C (*LMNA*)	0.16	5.5 × 10 ^−9^
14‐3‐3 protein theta (*YWHAQ*)	0.18	4.3 × 10^−8^
Thioredoxin related transmembrane protein 1 (*TMX1*)	0.28	1.1 × 10^−4^
60S ribosomal protein L12 (*RPL12*)	0.32	7.2 × 10^−4^
Chromosome 7 open reading frame 50 (*C7orf50*)	0.33	.001
X‐prolyl aminopeptidase 1 (*XPNPEP1*)	0.39	.006
Pleckstrin (*PLEK*)	0.44	.02
Small nuclear ribonucleoprotein F (*SNRPF*)	0.46	.03
Splicing factor 3a subunit 2 (*SF3A2*)	0.46	.03
Alanyl‐tRNA synthetase (*AARS*)	0.48	.04
Proteasome 26S, non‐ATPase regulatory subunit 2 (*PSMD2*)	0.48	.04

Protein (*GENE*) upregulated at day 7	FC D7/D0	*P*‐value
C‐type lectin domain family 3 member B (*CLEC3B*)	5.3	2.9 × 10^−5^
Coatomer subunit gamma (*COPG)*	4.6	2.3 × 10^−4^
Aldehyde dehydrogenase family 16 member A1 (*ALDH16A1*)	4	.002
ATPase H+ transporting V1 subunit A (*ATP6V1A*)	3.8	.003
Inter‐alpha‐trypsin inhibitor heavy chain 4 (*ITIH4*)	3.7	.004
Hsc70‐interacting protein (*ST13*)	3.2	.02
Inter‐alpha‐trypsin inhibitor heavy chain 3 (*ITIH3*)	3.2	.02
Dolichyl‐diphosphooligosaccharide protein glycosyltransferase subunit 2 (*RPN2*)	3.2	.02
Hemoglobin subunit alpha (HBA)	3	.03

*Notes:* Two additional proteins were significantly downregulated and 1 was significantly upregulated, but remained unidentified. Parentheses: official gene names according to the Hugo Gene Nomenclature Committee (https://www.genenames.org/).

Abbreviation: FC, fold change.

**FIGURE 1:**
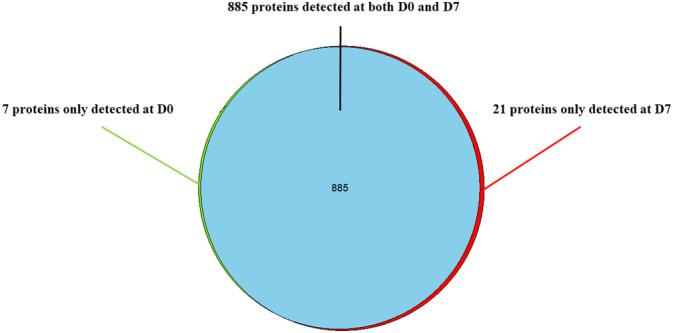
Venn diagram depicting proteins detected at both day 0 and day 7 (n = 885), detected only at day 0 but none of the dogs at day 7 (n = 7), and detected only at day 7 but none of the dogs at day 0 (n = 21)

A pitfall of retaining only proteins detected in over 50% of samples in each group is that proteins detected only at 1 time point but not at the other are excluded. Since these proteins could be biologically relevant, we further investigated them and displayed the results in Figure 1. We were able to identify 7 proteins that were detected only in the pre‐SFN samples, and 21 proteins detected only in the post‐SFN samples (Table 3).

**TABLE 3:** Proteins detected only presulforaphane (left column), or only postsulforaphane (right column)

Protein (*GENE*) detected only at day 0	Protein (*GENE*) detected only at day 7
Nucleolar protein (*NOP14*)	DExD/H‐box helicase 58 (*DDX58*)
Eukaryotic initiation factor 4A‐I (*EIF4A1*)	Serine/threonine kinase receptor associated protein (*STRAP*)
H3 histone family member 3B (*H3F3B*)	Myosin heavy chain‐1 (*MYH1*)
Copine 1 (*CPNE1*)	Exosome component 7 (*EXOSC7*)
NAD(P)H‐hydrate epimerase (*APOA1BP*)	MutS homolog 6 (*MSH6*)
Chromosome 7 C18orf25 homolog (*C7H18orf25*)	Chemerin chemokine‐like receptor 1 (*CMKLR1*)
	Complement 6 (*C6*)
	B cell receptor associated protein 31 (*BCAP31*)
	Penta‐EF‐hand domain containing 1 (*PEF1*)
	Host cell factor C1 (*HCFC1*)
	Ubiquitin like modifier activating enzyme 3 (*UBA3*)
	Signal sequence receptor subunit 3 (*SSR3*)
	Metaxin 1 (*MTX1*)
	Cytochrome b (*MT‐CYB*)
	Tubulin alpha 1a (*TUBA1A*)
	Centrosomal protein 72 (*CEP72*)
	Chromodomain helicase DNA binding protein 9 (*CHD9*)
	Taste receptor type 1 member 2 (*TAS1R2*)

*Notes:* One additional protein was only detected at day 0 and 3 only at day 7, but remained unidentified. Parentheses: official gene names according to the Hugo Gene Nomenclature committee.

### 3.3 Mass spectrometry: Intermediate/large B‐cell lymphoma at day 7 vs day 0

Due to the heterogeneity of our population of dogs in regard to their lymphoma phenotype, a subanalysis was performed that included only dogs with intermediate/large cell B‐LSA (n = 4, dogs #3, 4, 5, 6; dog #7 was excluded due to possible concurrent T‐zone LSA). Statistical analysis discovered 23 proteins with significant FC_B_ between D7 and D0 (*P* < .05). Among these, 10 were significantly downregulated at D7 (median FC_B_ D7/D0 = 0.16; range, 0.06‐0.27), and 13 were significantly upregulated at D7 (median FC_B_ D7/D0 = 4.3; range, 3.2‐5.3). After searching the raw data against Uniprot Canine and *Homo sapiens* database, 22 of these 23 proteins were precisely identified and the results are presented in Table 4. Of note, 7 of these proteins were also identified as significantly up‐ or down‐regulated when all dogs were analyzed together.

**TABLE 4:** Characteristics of 22 proteins significantly downregulated (n = 10) or upregulated (n = 12) after a week of oral supplementation with sulforaphane in 4 dogs with intermediate/large B‐cell multicentric lymphoma

Protein (*GENE*) downregulated at day 7	FC_B_ D7/D0	*P*‐value
**Thioredoxin related transmembrane protein 1 (*TMX1*)**	0.06	6.7 × 10 ^−11^
RRM domain‐containing protein (*N/A*)	0.07	1.4 × 10 ^−9^
ATP‐synt_C domain‐containing protein (*ATP5MC3*)	0.09	3.2 × 10^−8^
**tRNA‐splicing ligase RtcB homolog (*RTCB*)**	0.11	1.1 × 10^−6^
**Lamin A/C (*LMNA*)**	0.15	2.5 × 10^−5^
**Proteasome 26S, non‐ATPase regulatory subunit 2 (*PSMD2*)**	0.17	2.8 × 10^−4^
ArfGAP with FG repeats 1 (*MFF*)	0.19	4.8 × 10^−4^
Cytochrome c1 (*CYC1*)	0.24	5.7 × 10^−3^
Y‐box binding protein 1 (*YBX1*)	0.26	9.4 × 10^−3^
NADH dehydrogenase [ubiquinone] 1 alpha subcomplex subunit 8 (*NDUFA8*)	0.27	.01

Protein (*GENE*) upregulated at day 7	FC_B_ D7/D0	*P*‐value
ERH, mRNA splicing and mitosis factor (*ERH*)	5.3	7.3 × 10^−4^
Superoxide dismutase [Cu‐Zn] (*SOD1*)	5.3	7.3 × 10^−4^
**Dolichyl‐diphosphooligosaccharide—protein glycosyltransferase subunit 2 (*RPN2*)**	5.3	7.3 × 10^−4^
Small nuclear ribonucleoprotein Sm D3 (*SNRPD3*)	5.2	8.9 × 10^−4^
**Aldehyde dehydrogenase family 16 member A1 (*ALDH16A1*)**	4.9	1.5 × 10^−3^
Dyskerin pseudouridine synthase 1 (*DKC1*)	4.6	2.6 × 10^−3^
IRG‐type G domain‐containing protein (*N/A*)	4.3	4.7 × 10^−3^
Ig‐like domain‐containing protein (*N/A*)	3.9	.01
Methylosome subunit pICln (*CLNS1A*)	3.9	.01
**ATPase H+ transporting V1 subunit A (*ATP6V1A*)**	3.7	.01
Creatine kinase B‐type (*CKB*)	3.7	.01
Myo‐inositol oxygenase (*N/A*)	3.2	.04

*Notes:* One additional protein was significantly upregulated but remained unidentified. Parentheses: official gene names according to the Hugo Gene Nomenclature Committee (https://www.genenames.org/). Bold: proteins also significantly up‐ or down‐regulated when all dogs were compared together pre/post sulforaphane.

Abbreviations: FC, fold change; N/A, not applicable.

Finally, within the intermediate/large cells B‐LSA dogs, 3 proteins were detected only in the pre‐SFN samples and 7 proteins were detected only in the post‐SFN samples. Two of these proteins had also previously been detected at only on time point in the “all dogs” analysis: DExD/H‐box helicase 58, and NAD(P)H‐hydrate epimerase.

### 3.4 Mass spectrometry: Individual dogs at day 7 vs day 0

We then compared the differential expression of proteins before and after SFN administration in each dog. Of the 915 initial proteins, the number of proteins expressed at a level at least twice as low at D7 (FC D7/D0 < 0.5) or twice as high at D7 (FC D7/D0 > 2) ranged from 199 to 648 depending on the dog. As shown in Table 5, 3 dogs had more proteins upregulated at D7, while 3 dogs had more proteins downregulated at D7. Ras‐related C3 botulinum toxin substrate 1 was the protein upregulated to the highest extent for dog #1 and #3. Otherwise the proteomic changes were unique to each dog (Table 6).

**TABLE 5:** Number of proteins upregulated and downregulated by a least 2 folds for each dog after 7 days of oral supplementation with sulforaphane

Dog #	1	2	3	4	5	6	7
# of proteins retained	299	199	614	478	478	648	211
# (%) of proteins downregulated at D7	266 (89)	65 (33)	545 (89)	423 (89)	15 (3)	10 (2)	1 (0.5)
# (%) of proteins upregulated at D7	33 (11)	134 (67)	69 (11)	55 (11)	463 (97)	638 (98)	210 (99.5)

*Notes:* Proteins with an FC D7/D0 < 0.5 were considered to be downregulated at D7. Proteins with an FC D7/D0 > 2 were considered to be upregulated at D7.

**TABLE 6:** Characteristics of the most downregulated and most upregulated proteins for each dog after supplementation with sulforaphane

Dog #	Protein (*GENE*) most downregulated at D7	Protein (*GENE*) most upregulated at D7
**1**	Hsc70‐interacting protein (*ST13*) FC D7/D0 = 0.01	Ras‐related C3 botulinum toxin substrate 1 (*RAC1*) FC D7/D0 = 21.9
**2**	Wiskott‐Aldrich syndrome (*WAS*) FC D7/D0 = 0.03	Inter‐alpha‐trypsin inhibitor heavy chain 3 (*ITIH3*) FC D7/D0 = 21.4
**3**	Calcium‐transporting ATPase (*ATP2A3*) FC D7/D0 = 0.03	Ras‐related C3 botulinum toxin substrate 1 (*RAC1*) FC D7/D0 = 57.5
**4**	Uncharacterized protein FC D7/D0 = 0.02	Uncharacterized protein FC D7/D0 = 13.7
**5**	Thioredoxin related transmembrane protein 1 (*TMX1*) FC D7/D0 = 0.04	Core histone macro‐H2A (*H2AFY2*) FC D7/D0 = 79.3
**6**	tRNA‐splicing ligase RtcB homolog (*RTCB*) FC D7/D0 = 0.02	Signal recognition particle subunit SRP68 (*SRP68*) FC D7/D0 = 60.2
**7**	Uncharacterized protein FC D7/D0 = 0.33	Potassium channel tetramerization domain containing 12 (*KCTD12*) FC D7/D0 = 93.4

*Notes:* Parentheses: official gene names according to the Hugo Gene Nomenclature committee.

Abbreviation: FC, fold change.

### 3.5 Immunoblots

**FIGURE 2:**
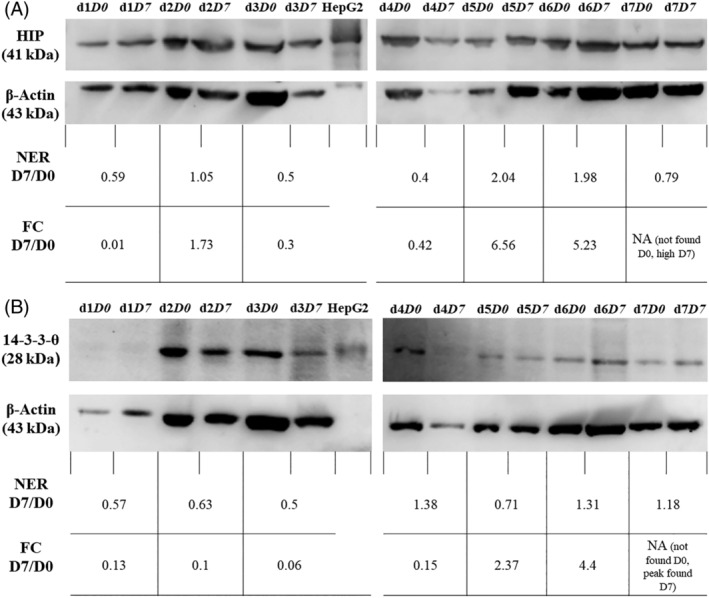
Immunoblots of lymph node samples for 7 dogs (d) with naïve multicentric lymphoma before (D0) and after (D7) supplementation with sulforaphane. The proteins HIP, A, and 14‐3‐3‐θ, B, were detected in all samples (top rows). The immunoblots for β‐actin (second rows) were used to normalize the band volume of HIP and 14‐3‐3‐θ. The normalized expression ratio at D7/D0 is the ratio of the normalized band volume at D7 vs D0 for each dog (third rows). For comparison, the corresponding fold change at D7 vs D0 previously determined by LC‐MS/MS is also annotated (bottom rows). FC D7/D0: Fold change at D7 vs D0 (mass spectrometry); NA: Not applicable; NER D7/D0: Normalized expression ratio at D7 vs D0 (immunoblot)

Two proteins were selected for validation of the LC‐MS/MS by immunoblots: heat shock 70 interacting protein (HIP) and 14‐3‐3 protein theta (14‐3‐3‐θ). As shown in Figure 2, the presence of HIP and 14‐3‐3‐θ in the lymph nodes samples of all dogs was confirmed. The NER D7/D0 was calculated for both proteins in each dog as described in the material and methods section. Changes in the expression level of HIP and 14‐3‐3‐θ after supplementation with SFN was largely concordant between the immunoblot results (NER D7/D0) and the previous LC‐MS/MS results (FC D7/D0). The only discordant result occurred for the 14‐3‐3‐θ protein in dog #4 (upregulated at D7 based on immunoblot, downregulated based on LC‐MS/MS), and dog #5 (downregulated at D7 based on immunoblot, upregulated based on LC‐MS/MS).

### 3.6 Gene set enrichment analysis

We generated a ranked list of genes from the LC‐MS/MS abundance ratio data as described in the Materials and Methods section. As some canine proteins were not encoded by a gene with a human ortholog, only 514 genes were included in the reference signature. Out of a total of 5917 gene sets from the GO database, 5052 were filtered out after restricting to our data set. Therefore, a total of 865 gene sets were used in the analysis. The median number of genes per set was 21 (range, 10‐155).

**FIGURE 3:**
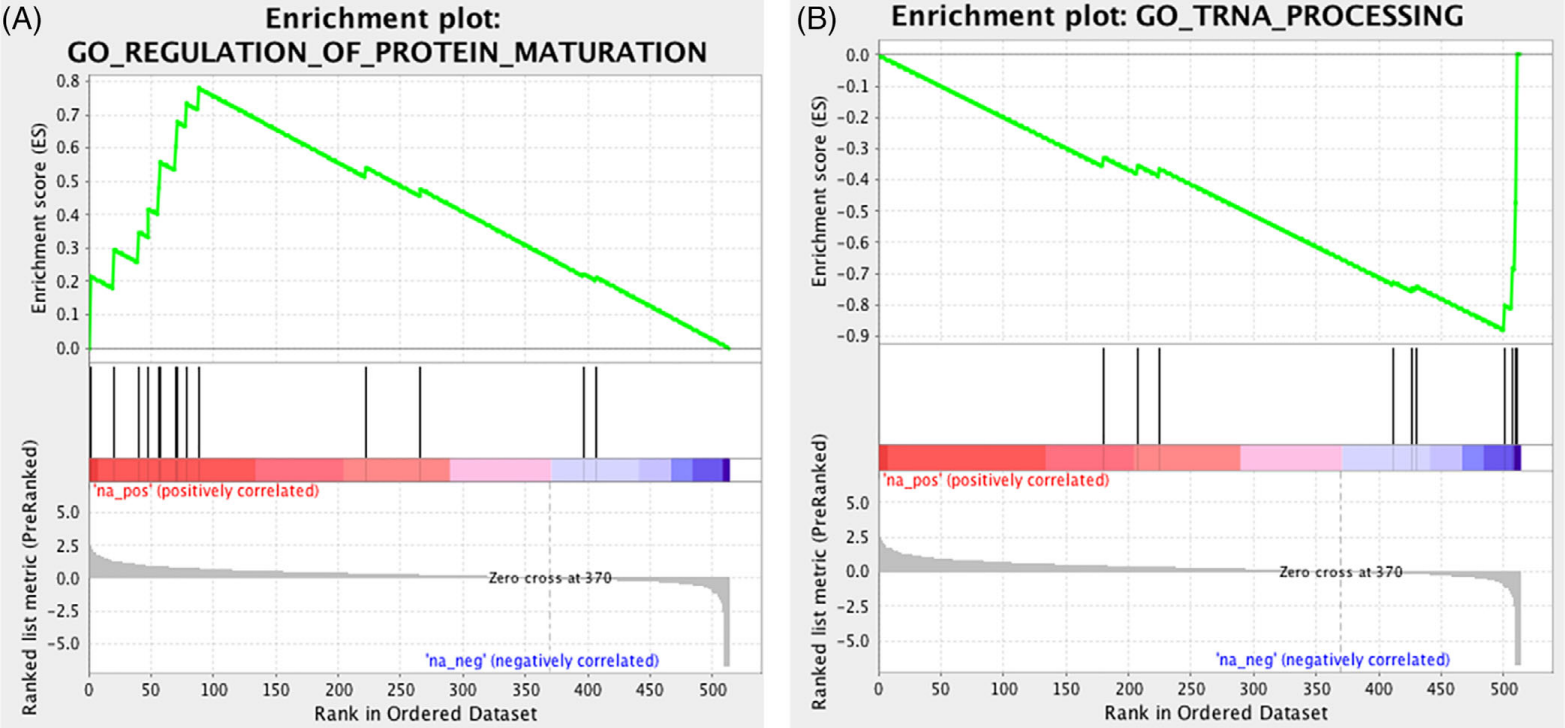
Enrichment plots of the most upregulated gene set (A, “regulation of protein maturation”) and the most downregulated gene set (B, “tRNA processing”) across all dogs postsulforaphane. The middle colored scale is a visual depiction of the ranked list of genes: the redder and more to the left, the most upregulated are the genes in the reference signature, the bluer and more to the right, the most downregulated are the genes in the reference signature. The vertical bars (“hits”) immediately above the color scale indicate where the genes of the gene set appear in the reference signature. The top portion of the plot displays the running enrichment score for the gene set as the analysis walks down the ranked list of genes of the reference signature. The enrichment score for a gene set is the score furthest from zero (peak of the plot for an upregulated gene set, bottom of the plot for a downregulated gene set). The enrichment score reflects the degree to which the genes contained in a gene sets are overrepresented on 1 side or the other of the reference signature

Overall, 720/865 gene sets (83.2%) were upregulated at D7, and 145/865 (16.8%) were downregulated at D7. The enrichment plot for the most upregulated gene set (“regulation of protein maturation”) and the most downregulated gene set (“tRNA processing”) are presented in Figure 3.

**FIGURE 4:**
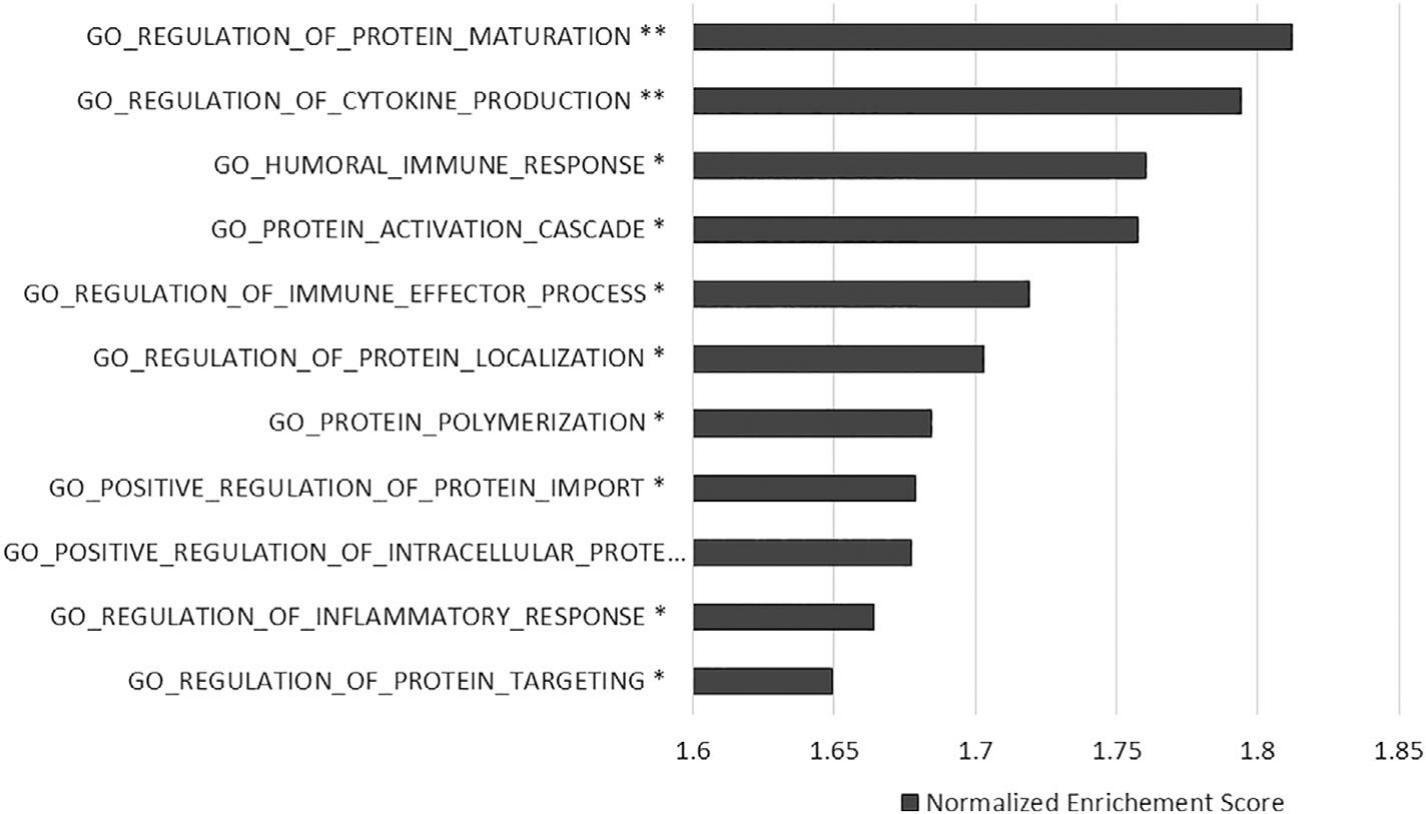
Bar graph displaying the normalized enrichment score of 11 gene sets significantly upregulated across all dogs at after sulforaphane. The gene sets, whose names appear on the left of each bar, are composed of genes annotated by the Gene Ontology (GO) term. Asterisks denote statistical significance as follows: *P < .01; **P < .001

When only including gene sets with a *P*‐value <.01 and a FDR *q*‐value <0.1, we identified 11 nonredundant gene sets (1.3%) significantly upregulated at D7. These gene sets included 2 main clusters: genes associated with immune response, and genes associated with protein maturation and transport, as detailed in Figure 4. Only 1 (0.1%) nonredundant gene set was significantly downregulated at D7. This gene set GO annotation was “tRNA processing” and the normalized enrichment score (NES) was −2.02 (*P* < .001).

## 4 DISCUSSION

The study presented here investigates the impact of supplementation with SFN in cancer‐bearing dogs. The results herein show that oral supplementation with twice daily SFN for 1 week was well tolerated in dogs with treatment‐naïve multicentric LSA, and induced pronounced changes in the expression level of several hundred proteins. Proteins and gene sets impacted by supplementation with SFN were frequently involved in regulation of innate and adaptive immunity, response to oxidative stress, gene transcription, apoptosis, and protein transport, maturation and ubiquitination.

Sulforaphane supplementation was well tolerated in this study. Most dogs experienced AEs, but many were noted prior to the start of the study and the most severe events occurred in dogs that experienced LSA progression. Therefore, none of the AEs were considered likely related to SFN; however, SFN AEs causality is possible. No dog demonstrated a clinical response after a week of supplementation with SFN. Although SFN has a major impact on cancer cell growth and survival in vitro,^16^, ^17^, ^18^, ^19^, ^20^, ^40^ and slows the growth of certain tumor xenografts,^20^, ^21^ a measurable gross benefit to SFN‐single‐agent therapy in patients with naturally occurring cancer has not been reported.

When all dogs were considered, 24 proteins were significantly up‐ or down‐regulated after supplementation with SFN. We found that, except for alanyl‐tRNA synthetase, all these proteins have been studied in relation to the pathogenesis or prognosis of various human malignancies including renal, liver, colorectal, pancreatic, pulmonary, head and neck, breast, and ovarian cancer.^41^, ^42^, ^43^ To our knowledge, none of these proteins have previously been investigated in veterinary oncology.

Thioredoxin related transmembrane protein 1 is involved in cellular response to oxidative stress and resistance to doxorubicin, cisplatin and etoposide in human T‐cell leukemia cell lines.^44^ We found this protein to be significantly downregulated after SFN (FC D7/D0 = 0.3). Increased reactive oxygen species production is a key mechanism of SFN tumor suppression. In human prostate cancer cells, SFN led to apoptosis by depleting GSH levels and increasing oxidative stress.^9^ The activity of thioredoxin and glutathione S‐transferase in lung cancer cells lines was also inhibited by covalent binding of SFN to their cysteine residues.^23^ To what extent alterations of cellular tolerance to oxidative stress is involved in SFN‐induced tumor cell death remains to be elucidated, as well as and potential interaction of SFN with reactive oxygen species‐producing chemotherapies such as doxorubicin.

Proteasome 26S non‐ATPase regulatory subunit 2 was significantly downregulated after supplementation with SFN (FC D7/D0 = 0.5). Sulforaphane can directly bind to and inhibit 26S and 20S proteasome subunits, leading to cell cycle arrest and apoptosis in multiple myeloma cell lines.^23^ Since drugs targeting the proteasome 26S subunit, such as bortezomib, have been used successfully in combination with various chemotherapeutics and HDACi to treat myeloma and various types of LSA in human,^45^, ^46^ the downregulation of proteasome subunits after a week of supplementation with SFN could represent an important anticancer strategy to be explored in future studies.

Numerous proteins influenced by SFN in this study are involved in regulation of innate or adaptive immunity. For example, the proteins C‐type lectin domain family 3 member B and inter‐alpha‐trypsin inhibitor heavy chain (involved in response to TGF‐β and IL‐6) were significantly upregulated across all dogs following supplementation with SFN. In addition, 7 proteins that were not detected in any dogs at D0, but in ≥1 dog at D7 (DExD/H‐box helicase 58, serine/threonine kinase receptor associated protein, exosome component 7, MutS homolog 6, chemerin chemokine‐like receptor 1, Complement 6 and B‐cell receptor associated protein 31) are involved in regulation of cytokine production, macrophage chemotaxis, complement receptor signaling, formation of the membrane attack complex, MHC class I binding, immunoglobulin class switch, and somatic hypermutation.^41^, ^42^, ^43^, ^47^, ^48^, ^49^ Moreover, 3 upregulated gene sets are also involved in regulation of the immune system (Figure 4). Immunotherapy has revolutionized the treatment of some human LSA^50^, ^51^ and encouraging results have recently been reported in dogs with LSA.^5^, ^52^, ^53^ Therefore, the relevance of SFN impact on the immune system should be investigated further.

Sulforaphane interacts with several cancer pathways including PI3K/AKT and MAPK/MEK/ERK in colon and prostate cancer, leukemia and multiple myeloma cell lines.^9^, ^19^, ^20^, ^21^, ^35^ Interestingly, several proteins identified in this study are known to interplay with these pathways. For example, lamin A/C and pleckstrin, both of which are positively associated with the PI3K/AKT pathway,^41^, ^42^, ^43^, ^47^, ^54^, ^55^ were significantly downregulated following supplementation with SFN in this study (FC D7/D0 = 0.2 and 0.4, respectively). The protein potassium channel tetramerization domain containing 12, which has been shown to inhibits the ERK pathway,^56^ was upregulated >2‐fold (up to 93‐fold) in 3 dogs following SFN. These proteins could later prove pivotal to SFN‐induced tumor prevention or suppression.

We did not set inclusion criteria regarding LSA phenotype. As a result, our population of dogs was heterogeneous and included 3 B‐LSA, 2 T‐LSA, and 1 B‐LSA with a possible concurrent T‐zone LSA. Since this could have had a meaningful impact on the results, a subanalysis that included only the 4 cases with intermediate/large cells B‐LSA was performed. Within this more homogeneous LSA population analysis, we found that 23 proteins were significantly up or downregulated post‐SFN, of which 7 had already been identified as significantly impacted by SFN when all dogs were analyzed together, including the previously discussed proteins thioredoxin related transmembrane protein 1 and proteasome 26S subunit 2. Moreover, in agreement with the findings from the “all dogs” analysis, several of these proteins were involved in regulation of innate or adaptive immunity (ex: Ig‐like domain‐containing protein, IRG‐type G domain‐containing protein) or response to oxidative stress (ex: superoxide dismutase, aldehyde dehydrogenase).

The proteins HIP and 14‐3‐3‐θ were chosen to validate the mass spectrometry results as they were among those proteins exhibiting different expression between D0 and D7 according to the LC‐MS/MS results. Additionally, the antibodies targeting these proteins were predicted to be cross‐reactive with the canine protein according to the manufacturer. Based on LC‐MS/MS, HIP was downregulated at D7 in dogs # 1, #3, #4 while it was upregulated in dogs #2, #5, #6. These results were confirmed in all dogs via immunoblots. Moreover, 14‐3‐3‐θ was downregulated at D7 in dogs #1, #2, #3, #4, and upregulated in dogs #5 and 6. The results were confirmed in 3 dogs via immunoblots, while for 2 dogs (#4 and #5), the LC‐MS/MS and immunoblots results were reversed. The cause of the discordant results between the LC‐MS/MS and immunoblots for 1 of the proteins in 2 dogs is unclear. The authors suspect a likely human error at 1 of the steps of the immunoblots for these dogs (protein quantification or dilution, electrophoresis, transfer on nitrocellulose membrane, antibody binding) since in our opinion, a human error is more likely to have occurred during these experiments than during mass spectrometry which is a more automatized and sensitive analysis.

To our knowledge, 5 proteomic studies of LSA in dogs have been published.^57^, ^58^, ^59^, ^60^, ^61^ In 2 studies, the mass‐to‐charge ratio (m/z) of the protein peaks or the peptide mass profile were reported, however the identity of the proteins was not available.^58^, ^61^ In the other studies, the proteins differentially expressed between dogs with LSA and healthy dogs included haptoglobin, C‐reactive protein, α2‐macroglobulin, apolipoprotein A1 precursor, inter α‐trypsin inhibitor, and several others.^57^, ^59^, ^60^ Among the dozens of proteins of interest identified in the current study, only 3 proteins have been reported in previous proteomic studies in dogs with LSA. Inter‐alpha‐trypsin inhibitor was detected by Atherthon et al using LC–MS/MS (ITIH3 and ITIH4 were significantly upregulated post‐SFN).^57^ Proteins α2 macroglobulin and apolipoprotein A1 were also both detected by Atherthon et al, and by us in the GSEA (both were genes from the “coagulation” gene set, significantly upregulated post‐SFN).

This study is limited by the small number of dogs included, as well as the heterogeneity in lymphoma phenotype. However, the primary goals of this study was to determine if SFN had any impact at all on the proteome of cancer‐bearing dogs and identify promising protein targets to be investigated in follow‐up studies. In addition, we cannot exclude the possibility that the changes noted in the expression level of various proteins occurred for reasons unrelated to supplementation with SFN. Factors such as physiologic variation in protein production and catabolism, sampling of different lymph nodes areas at D0 and D7, or metabolic changes due to LSA progression during the week of supplementation with SFN might be considered. Finally, among all the proteins of interest discussed above, only the results obtained for the proteins HIP and 14‐3‐3‐θ have been validated with 2 independent methodologies (LC‐MS/MS and immunoblots), while the others have been evaluated by LC‐MS/MS. Therefore, our findings regarding the influence of SFN on multiple proteins and oncogenic pathways in dogs with LSA remain preliminary.

## CONFLICT OF INTEREST DECLARATION

Authors declare no conflict of interest.

## OFF‐LABEL ANTIMICROBIAL DECLARATION

Authors declare no off‐label use of antimicrobials.

## INSTITUTIONAL ANIMAL CARE AND USE COMMITTEE (IACUC) OR OTHER APPROVAL DECLARATION

Approval of the Oregon State University (OSU) IACUC (Animal Care and Use Protocol number 4893, approved on 02/03/2017).

## HUMAN ETHICS APPROVAL DECLARATION

Authors declare human ethics approval was not needed for this study.
